# Serum 1,5-anhydroglucitol when used with fasting plasma glucose improves the efficiency of diabetes screening in a Chinese population

**DOI:** 10.1038/s41598-017-12210-z

**Published:** 2017-09-20

**Authors:** Lingwen Ying, Xingxing He, Xiaojing Ma, Yun Shen, Hang Su, Jiahui Peng, Yufei Wang, Yuqian Bao, Jian Zhou, Weiping Jia

**Affiliations:** Department of Endocrinology and Metabolism, Shanghai Jiao Tong University Affiliated Sixth People’s Hospital; Shanghai Clinical Center for Diabetes; Shanghai Diabetes Institute; Shanghai Key Laboratory of Diabetes Mellitus, Shanghai, 200233 China

## Abstract

Serum 1,5-anhydroglucitol (1,5-AG) levels can not only accurately reflect the mean blood glucose over the previous 1–2 weeks in diabetic patients but also offers the advantage of representing postprandial glucose. To evaluate the clinical significance of 1,5-AG in diabetes detection, especially when used in combination with fasting plasma glucose (FPG), a total of 3098 participants at high risk for diabetes (1467 men, 1631 women) were enrolled. A total of 1471 (47.5%) participants were diagnosed with diabetes, and the mean 1,5-AG level in the diabetic group was significantly lower than that in non-diabetic group [12.5 (7.8–17.5) μg/mL *vs*. 20.5 (15.3–26.4) μg/mL, *P* < 0.001]. The optimal cut-off point was 15.9 μg/mL, for which the sensitivity, specificity, and area under the curve (AUC) were 69.2%, 72.3%, and 0.781, respectively. For the combination of FPG and 1,5-AG, the sensitivity, specificity, and AUC improved to 82.5%, 83.5%, and 0.912, respectively. This method helped 75.8% of the participants avoid an oral glucose tolerance test (OGTT), reducing the need to carry out the OGTT by 43.9% compared to the use of the FPG criterion only. In conclusion, the addition of FPG to serum 1,5-AG improves the efficiency of diabetes screening in the Chinese population.

## Introduction

Diabetes mellitus has become a major problem affecting human health. According to the prediction of International Diabetes Federation (IDF), there will be 552 million individuals suffering from diabetes worldwide by 2030, and most patients will be in low-income countries^1^. An epidemiological survey conducted in 2013 also showed that the undiagnosed diabetes rate was estimated to be 6.9% in the Chinese population^2^. Patients usually have chronic hyperglycemia for quite a long time before being diagnosed with diabetes. Moreover, diabetes complications caused by hyperglycemia, such as diabetic angiopathy and diabetic nephropathy, significantly affect patients’ quality of life and the clinical prognosis^3,4^. Thus, early detection, early diagnosis, and early intervention for patients with diabetes are of critical importance.

The 75-g oral glucose tolerance test (OGTT) serves as the “gold standard” in the clinical diagnosis of diabetes^5^. However, due to its complicated procedure and susceptibility to a variety of factors, the application of OGTT is limited^6^. Therefore, the fasting plasma glucose (FPG), rather than OGTT, is more commonly used in diabetes screening. However, it was reported that the patients with diabetes in Europe were featured with isolated fasting hyperglycemia^7,8^, whereas most Chinese diabetic patients presented with isolated postprandial hyperglycemia^9^. Without an OGTT, a majority of individuals with isolated postprandial hyperglycemia in the Chinese population would be missed if screened based on only FPG detection.

1,5-Anhydroglucitol (1,5-AG), a six-carbon chain monosaccharide, can accurately reflect the mean blood glucose level over the previous 1–2 weeks in diabetic patients^10,11^. Previous research also demonstrated that serum 1,5-AG offers an advantage of representing postprandial glucose^12^. Furthermore, some studies have investigated whether serum 1,5-AG can be used for diabetes screening. The present study aimed to evaluate the clinical significance of 1,5-AG in diabetes screening, especially when combined with FPG, with respect to its ability to facilitate early diagnosis and intervention in diabetes patients.

## Results

### Clinical characteristics of study participants

The characteristics of the 3098 subjects enrolled in the present study, including 1471 (47.5%) with diabetes and 1627 (52.5%) without diabetes, are shown in Table 1. The numbers of participants with isolated postprandial hyperglycemia, isolated elevated glycated hemoglobin A_1c_ (HbA_1c_), and elevated 2-hour postload plasma glucose (2hPG) and HbA_1c_ along with a FPG <7.0 mmol/L were 438 (29.8%), 111 (7.5%), and 227 (15.4%), respectively. These results indicated that 52.8% (776/1471) of participants with diabetes would be missed in screening based on merely FPG detection. Age, systolic blood pressure (SBP), body mass index (BMI), HbA_1c_, FPG, and 2hPG levels were significantly higher in patients with diabetes than in participants without diabetes in both genders (all *P* < 0.05). In addition, in female participants, the diastolic blood pressure (DBP) was significantly higher in those with diabetes than in those without diabetes (*P* < 0.05).

**Table 1:** Characteristics of the study participants.

Variables	Total	Male			Female		
		Non-DM	DM	*P*	Non-DM	DM	*P*
*n* (%)	3098	698	769	—	929	702	—
Age (years)	54 (42–61)	50 (39–60)	54 (45–61)	<0.001	51 (36–60)	58 (51–63)	<0.001
BMI (kg/m^2^)	24.3 (22.2–26.7)	24.5 (22.4–26.8)	25.3 (23.4–27.5)	<0.001	23.3 (21.4–25.7)	24.2 (22.1–26.7)	<0.001
Systolic blood pressure (mmHg)	130 (119–142)	130 (120–140)	132 (121–143)	0.023	125 (114–139)	135 (123–147)	<0.001
Diastolic blood pressure (mmHg)	80 (72–86)	80 (74–88)	82 (75–88)	0.067	76 (68–83)	80 (73–86)	<0.001
1,5-AG (μg/mL)	16.5 (11.2–23.1)	22.2 (16.7–28.5)	11.3 (6.8–16.4)	<0.001	19.3 (14.4–25.1)	13.6 (8.8–18.5)	<0.001
HbA_1c_ (%)	6.0 (5.6–6.5)	5.7 (5.4–5.9)	6.6 (6.2–7.2)	<0.001	5.6 (5.3–5.9)	6.5 (6.1–6.9)	<0.001
HbA_1c_ (mmol/mol)	38 (34–44)	35 (32–37)	45 (40–51)	<0.001	34 (31–37)	44 (39–48)	<0.001
FPG (mmol/L)	6.0 (5.4–6.9)	5.6 (5.2–6.0)	7.0 (6.3–7.8)	<0.001	5.4 (5.0–5.8)	6.8 (6.1–7.5)	<0.001
2hPG (mmol/L)	9.9 (7.4–13.4)	8.0 (6.4–9.4)	13.9 (12.0–16.3)	<0.001	7.4 (6.1–8.8)	13.3 (11.8–15.6)	<0.001
FDR, *n* (%)	1372 (44.3)	268 (38.4)	329 (42.8)	0.049	421 (45.3)	354 (50.4)	0.023

Data were expressed as median (interquartile range), or *n* (%). Abbreviation: *1,5-AG*: 1,5-anhydroglucitol; *2hPG*: 2-hour postload plasma glucose; *BMI*: body mass index; *DM*: diabetes mellitus; *FDR*: first degree relative with diabetes; *FPG*: fasting plasma glucose; *HbA*_*1c*_: glycated hemoglobin A_1c_; *Non-DM*: non-diabetes mellitus.

**Figure 1:**
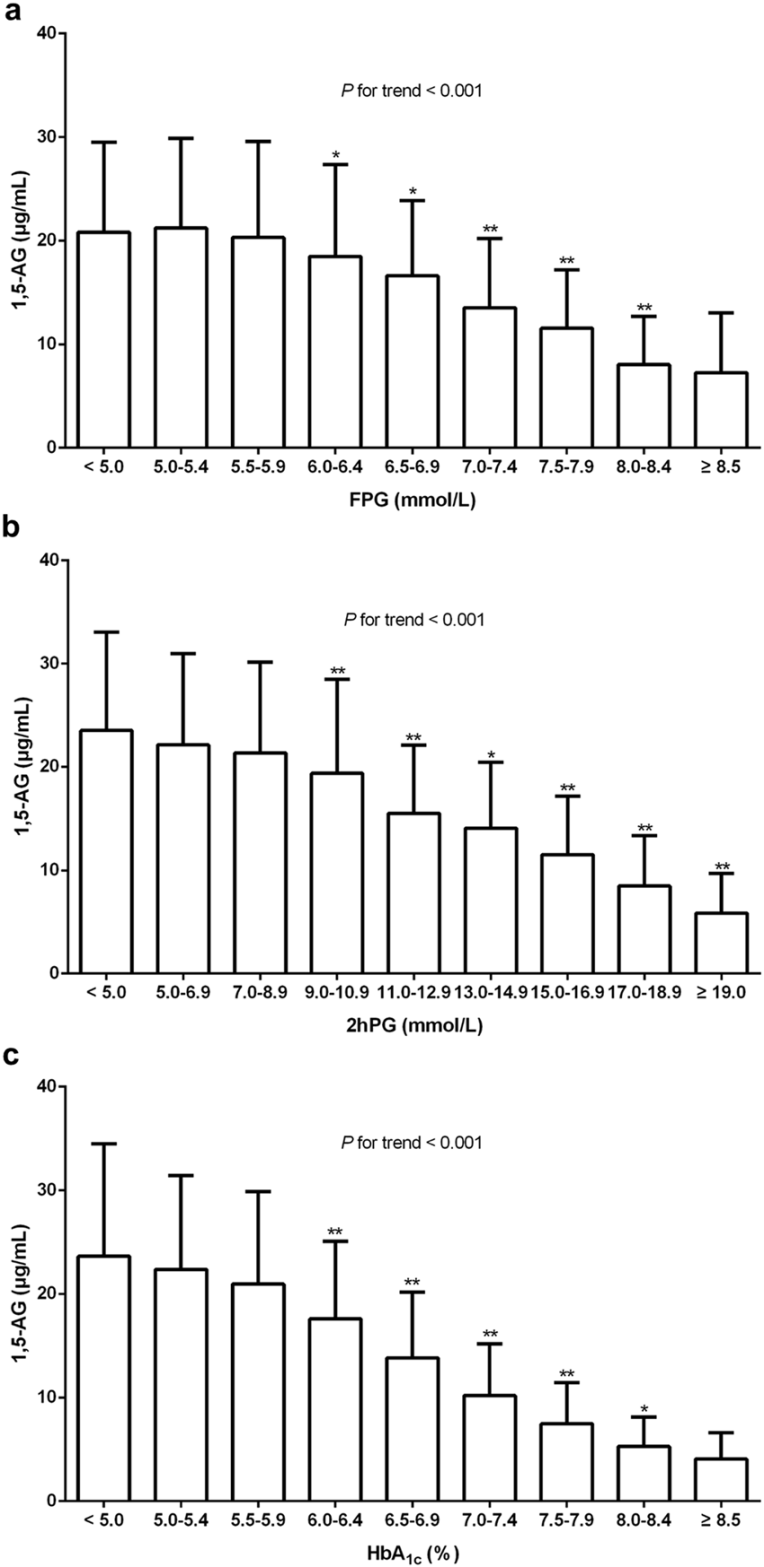
Characteristics of 1,5-anhydroglucitol (1,5-AG) levels in the total group (**a**) compared with different FPG levels; (**b**) compared with different 2hPG levels; (**c**) compared with different HbA_1c_ levels). ^*^ *P* < 0.05 versus the former group, ^**^ *P* < 0.01 versus the former group. Abbreviation: *2hPG*, 2-hour postload plasma glucose; *FPG*: fasting plasma glucose; *HbA*_*1c*_, glycated hemoglobin A_1c_.

The mean serum 1,5-AG level in the total group was 16.5 (11.2–23.1) μg/mL, and no significant difference in 1,5-AG levels was observed between males and females (*P* > 0.05). Furthermore, serum 1,5-AG levels in participants with diabetes were significantly lower than those in participants without diabetes [12.5 (7.8–17.5) μg/mL *vs*. 20.5 (15.3–26.4) μg/mL, *P* < 0.001]. As shown in Fig. 1, serum 1,5-AG levels decreased as the HbA_1c_, FPG, and 2hPG values increased. Further inter-group comparison analysis showed that serum 1,5-AG levels significantly and gradually declined when FPG, 2hPG, and HbA_lc_ reached 6.0 mmol/L, 9.0 mmol/L, and 6.0%, respectively, and stayed steady once the FPG was ≥8.5 mmol/L or HbA_1c_ ≥ 8.5%. Specifically, there was no plateau in 1,5-AG levels with changes in the 2hPG.

### Correlation analysis of serum 1,5-AG level

Spearman correlation analysis showed that the 1,5-AG level was negatively associated with FPG, 2hPG, HbA_1c_, and BMI (all *P* < 0.001). However, no significant correlation was observed between 1,5-AG and age, SBP, or DBP (all *P* > 0.05). Furthermore, multiple stepwise regression analysis with 1,5-AG as a dependent variable and FPG, 2hPG, HbA_1c_, BMI, and first degree relative with diabetes (FDR) as independent variables showed that HbA_1c_, 2hPG, and FDR were independently correlated with 1,5-AG levels (standardized *β* = −0.295, −0.298, and −0.061, respectively, all *P* < 0.001; Table 2).

**Table 2:** Multivariate regression analyses of factors associated with serum 1,5-AG levels.

Variables	Standardized *β*	*t*	*P*
2hPG	−0.298	−13.697	<0.001
HbA_1c_	−0.295	−13.530	<0.001
FDR	−0.061	−4.061	<0.001

Independent variables originally included: FPG, 2hPG, HbA_1c_, BMI, and FDR. Abbreviation: *1,5-AG*: 1,5-anhydroglucitol; *2hPG*:2-hour postload plasma glucose; *BMI*: body mass index; *FDR*: first degree relative with diabetes; *FPG*: fasting plasma glucose; *HbA*_*1c*_: glycated hemoglobin A_1c_.

### Serum 1,5-AG in diabetes screening

**Figure 2:**
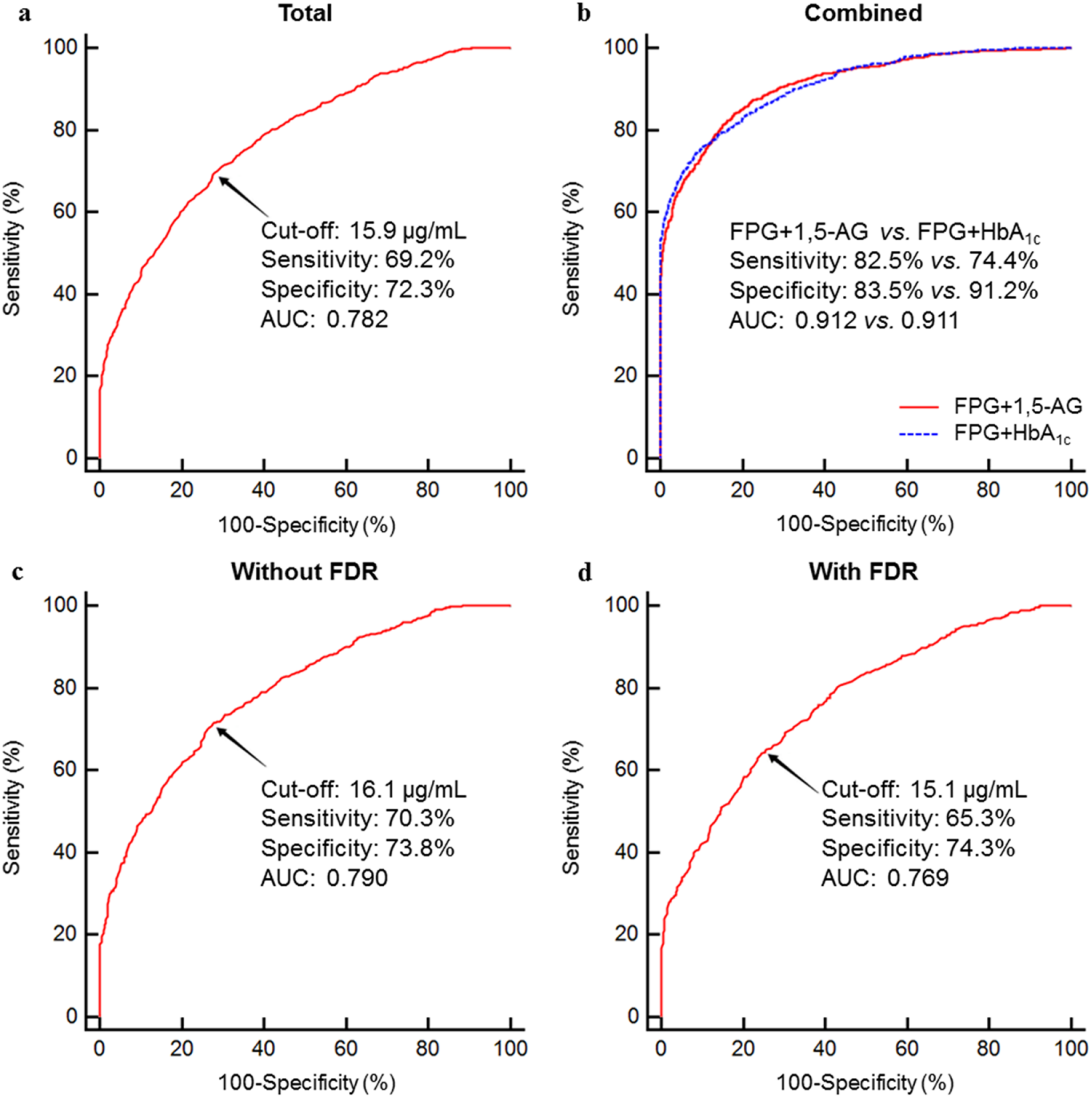
ROC curves for 1,5-anhydroglucitol (1,5-AG) ((**a**) in the total group; (**b**) in the total group with combined model; (**c**) in the non-FDR subgroup; (**d**) in the FDR subgroup) in the diagnosis of diabetes. For the combination of FPG with serum 1,5-AG, the sensitivity, specificity, and AUC were 82.5%, 83.5%, and 0.912, respectively. As for the combination of FPG and HbA_1c_, the sensitivity, specificity, and AUC were 74.4%, 91.2%, and 0.911, respectively. There was no significant difference in the AUCs (*P* > 0.05). However, the specificity and sensitivity differed significantly from each other (all *P* < 0.001). Abbreviation: *AUC*: area under the curve; *FDR*: first degree relative with diabetes; *FPG*: fasting plasma glucose; *HbA*_*1c*_: glycated hemoglobin A_1c_; *ROC*: receiver operating characteristic.

Figure 2a presents the receiver operating characteristic (ROC) curve for the use of serum 1,5-AG in detecting undiagnosed diabetes based on the 2010 American Diabetes Association (ADA) diabetes diagnostic criteria. The analysis demonstrated that the optimal serum 1,5-AG cut-off point for identification of diabetes was 15.9 μg/mL, with a sensitivity, specificity, positive predictive value (PPV), negative predictive value (NPV), and area under the curve (AUC) of 69.2% (95% confidence interval [*CI*]: 66.8–71.6%), 72.3% (95%*CI*: 70.1–74.5%), 69.4% (95%*CI*: 66.9–71.7%), 72.2% (95%*CI*: 70.0–74.4%), and 0.781 (95%*CI*: 0.766–0.796), respectively. For the combination of FPG with serum 1,5-AG, the sensitivity, specificity, and AUC further improved to 82.5% (95%*CI*: 80.4–84.4%), 83.5% (95%*CI*: 81.6–85.2%), and 0.912 (95%*CI*: 0.901–0.921), respectively. As for the combination of FPG and HbA_1c_, there was no significant difference in the AUC when compared with the combination of FPG and 1,5-AG (0.911 *vs*. 0.912, *P* > 0.05). At the same time, the specificity was 91.2%, while the sensitivity was merely 74.4%. Though the specificity of the combination of FPG and HbA_1c_ was superior (91.2% *vs*. 83.5%, *P* < 0.001), its sensitivity was significantly lower when compared with the combination of FPG and 1,5-AG (74.4% *vs*. 82.5%, *P* < 0.001; Fig. 2b).

The ROC models were further stratified based on the existence of a FDR, because of the significant interaction between FDR and serum 1,5-AG. The AUC for 1,5-AG did not differ significantly between the subgroups with and without a FDR [0.769 (0.745–0.791) *vs*. 0.790 (0.770–0.809), *P* = 0.202; Fig. 2c,d].

### Combination of FPG and serum 1,5-AG can guide the diagnosis of diabetes

Among the 3098 participants, 1068 participants had a FPG <5.6 mmol/L, and 695 had a FPG ≥ 7.0 mmol/L. These data indicated that about 43.1% (1335/3098) of participants required an OGTT for further diagnosis after screening based merely on FPG detection. Further analysis showed that, of the 1468 individuals with a serum 1,5-AG level ≤15.9 μg/mL, 1018 were diagnosed with diabetes (PPV 69.4% [95%*CI*: 66.9–71.7%], NPV 72.2% [95%*CI*: 70.0–74.4%]). Similarly, of the 1607 found to have a FPG ≥ 7.0 mmol/L and/or serum 1,5-AG level ≤15.9 μg/mL, 1157 were diagnosed with diabetes with an improved PPV of 72.0% (95%*CI*: 69.7–74.2%) and a NPV of 78.9% (95%*CI*: 76.8–81.0%). Conversely, of those found to have a FPG < 5.6 mmol/L and serum 1,5-AG level >15.9 μg/mL (*n* = 741), only 61 were diagnosed with diabetes.

**Figure 3:**
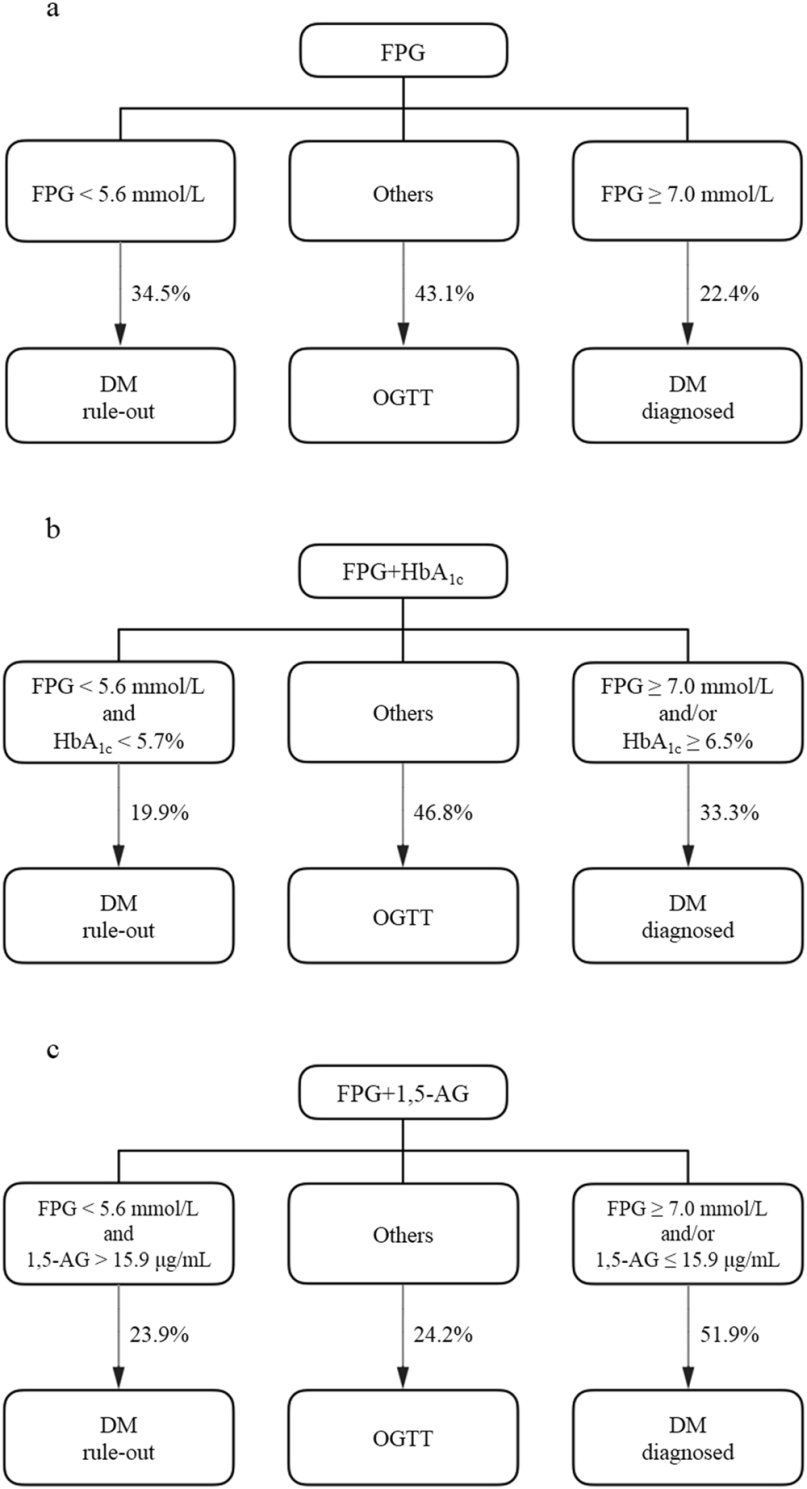
Screening strategies for detecting diabetes by an OGTT after use of (**a**) the FPG criteria, (**b**) the combination of FPG and HbA_1c_, or (**c**) the combination of FPG and 1,5-AG. The proportions of the study population in specific diagnostic categories were: (**a**) FPG < 5.6 mmol/L to exclude and FPG ≥ 7.0 mmol/L to diagnose diabetes, with 43.1% participants needing an OGTT to confirm the diagnose. The sensitivity, specificity, PPV, and NPV for this strategy were 47.3%, 100%, 100%, and 67.7%, respectively. (**b**) As for the combined criteria of FPG and HbA_1c_ criteria, FPG < 5.6 mmol/L and HbA_1c_ < 5.7% to exclude diabetes, and FPG ≥ 7.0 mmol/L and/or HbA_1c_ ≥ 6.5% to confirm diabetes, 46.8% participants needed an OGTT. The sensitivity, specificity, PPV, and NPV for this strategy were 70.2%, 100%, 100%, and 78.8%, respectively. (**c**) With the combined FPG and 1,5-AG criteria, which are FPG < 5.6 mmol/L and 1,5-AG > 15.9 μg/mL for exclusion of diabetes, and FPG ≥ 7.0 mmol/L and/or 1,5-AG ≤ 15.9 μg/mL for the diagnosis of diabetes, only 24.2% participants needed an OGTT. The sensitivity, specificity, PPV, and NPV for this strategy were 78.7%, 72.3%, 72.0%, and 78.9%, respectively. Abbreviations: *DM*: diabetes mellitus; *FPG*: fasting plasma glucose; *HbA*_*1c*_ *:* glycated hemoglobin A_1c_; *NPV*: negative predictive value; *OGTT*: oral glucose tolerance test; *PPV*: positive predictive value.

Thus, based on the FPG criteria only, 43.1% of the participants required an OGTT for identification of diabetes (5.6 mmol/L ≤ FPG < 7.0 mmol/L), with sensitivity, specificity, PPV, and NPV values of 47.3%, 100%, 100%, and 67.7%, respectively (Fig. 3a). While with the combined criteria of FPG and HbA_1c_, 46.8% of the subjects need an OGTT for further confirmation (Fig. 3b). With the combined criteria of FPG and 1,5-AG, diabetes could be ruled out when FPG < 5.6 mmol/L and 1,5-AG > 15.9 μg/mL, and individuals should be diagnosed with diabetes if they have either a FPG ≥ 7.0 mmol/L or serum 1,5-AG level ≤15.9 μg/mL, If neither of these criteria are met, OGTT is recommended to confirm the diagnosis (Fig. 3c). With the criteria above, the sensitivity, specificity, PPV, and NPV for the combination of FPG and 1,5-AG were 78.7%, 72.3%, 72.0%, and 78.9%, respectively. Therefore, with use of the combination of FPG and 1,5-AG, OGTT analysis could be avoided in 75.8% (2348/3098) of our study participants. In other words, use of the combination criteria would reduce the need to carry out OGTT by 43.9% in comparison with the use of the FPG criterion only.

## Discussion

Our study indicated that the combined use of FPG and serum 1,5-AG criteria can improve the diagnostic efficiency of diabetes screening in a Chinese population. With this approach, OGTT could be avoided in 75.8% of the participants, and thus, the need for OGTT was reduced by 43.9% compared to that when only the FPG criterion was used. Also, the optimal cut-off point for serum 1,5-AG in diabetes screening was 15.9 μg/mL.

Then, we further compare the predictive value of the combination of FPG and 1,5-AG with that of FPG and HbA_1c_. When compared with the combination of FPG and HbA_1c_, there’s no significant difference between the AUC of these two combined criteria. However, the sensitivity of the combination of FPG and 1,5-AG was higher than that of FPG and HbA_1c_, and the specificity was lower. As a screening method, the ability to predict the positive population (i.e. sensitivity) is more important than specificity when a certain screening efficiency is ensured. Thus, the combination of FPG and 1,5-AG is suitable for diabetic screening.

The level of 1,5-AG, a polyol in the blood, is stable in individuals, with little fluctuation and no diurnal difference. 1,5-AG is mainly reabsorbed through the renal tube and maintained at a high level in normal circumstances. When the blood glucose exceeds the renal glucose threshold, the absorption of 1,5-AG will be inhibited by glucose^13–15^. Yamanouchi *et al*.^16^ found that the plasma 1,5-AG level multiplied by the urinary glucose level was relatively constant, indicating that 1,5-AG is negatively correlated with blood glucose. Therefore, serum 1,5-AG measurement can both provide information about glycemic control and reflect short-term (over 1–2 weeks) glucose fluctuations^10,11^. In addition, another study conducted by Kim *et al*.^17^ showed that serum 1,5-AG might be a reliable glycemic index in mild-to-moderate renal dysfunction as well. Our study also found that the serum 1,5-AG level was significantly negatively correlated with FPG, 2hPG, and HbA_1c_ levels.

Previous studies have shown that glycemic distributions vary among different races. For example, in the European population, diabetes patients mainly present with increased FPG^7,8^, whereas in the Asian population, diabetes patients exhibit postprandial hyperglycemia^18^, which was also observed in the current study. It was reported that 1,5-AG is clearly superior to HbA_1c_ in the accessment of postprandial glucose levels, especially for patients with HbA_1c_ < 8%^17,18^. Similarly, the current study also found that the serum 1,5-AG levels correlated negatively with glucose levels, and the association with 2hPG was superior to the association of FPG with serum 1,5-AG. In addition, serum 1,5-AG was more sensitive for diabetes in cases with 2hPG ≥ 9.0 mmol/L or HbA_1c_ < 8.5%.

The current study, for the first time, indicates the combination of FPG and serum 1,5-AG can guide diabetes screening. Based on FPG criteria only, 43.1% of the study participants would require an OGTT for definitive diagnosis, whereas with the combined use of FPG with serum 1,5-AG for detecting diabetes, only 24.2% of the study participants would need an OGTT. These findings indicate that the number of subjects requiring an OGTT can be reduced by more than 40% based on the combined use of FPG and 1,5-AG criteria, making screening for diabetes more sensitive and convenient. Our results also showed that the optimal cut-off point for 1,5-AG for the detection of diabetes in our Chinese population was 15.9 μg/mL. Shirasaya *et al*.^19^ conducted a study in 891 Japanese subjects undergoing routine health examination and reported that the optimal cut-off point for 1,5-AG was 17.1 μg/mL. Another related study in Japan (with 77 enrolled outpatients) showed that a serum 1,5-AG concentration of 14.2 μg/mL would be the best cut-off point for diabetes detection^20^. Based on the results outlined above, the ideal cut-off point for serum 1,5-AG in diabetes screening in Asian populations might fall in the range of 14.2–17.1 μg/mL, and specific cut-off points for specific populations require further research. Interestingly, a study conducted by Wang *et al*.^21^ based on a community population in Nanjing (25.5% of the participants were diagnosed with diabetes), the optimal cut-off point for serum 1,5-AG was 11.18 μg/mL. As mentioned in the discussion part, the gender imbalance and small sample size may explain this discrepancy. Moreover, we further performed a subgroup analysis based on FDR and found no significant difference in the screening efficacy of 1,5-AG between individuals with FDR and those without FDR.

There are some limitations in the current study. First, this was a single-center study, and the results need to be confirmed in a larger multi-center study. Second, further prospective research is needed given the cross-sectional design of this study. In addition, the present study population was selected from outpatients at high risk of diabetes, and future research is needed to confirm the findings in a larger cohort of general population.

In conclusion, we confirmed that the serum 1,5-AG level offers the advantage of reflecting postprandial glucose. This might explain why the combination of FPG and serum 1,5-AG criteria improved the early detection of diabetes in our Chinese population. In addition, the use of both FPG and serum 1,5-AG criteria helps to determine whether an OGTT is needed, allowing 75.8% of the participants to avoid an OGTT.

## Materials and Methods

### Study population

A total of 3098 individuals (1467 men, 1631 women; age range 20–80 years) who underwent a 75-g OGTT in the outpatient clinic of the Department of Endocrinology and Metabolism of Shanghai Jiao Tong University Affiliated Sixth People's Hospital between January 2011 and March 2017 were enrolled in the study. All participants had no history of diabetes mellitus or impaired glucose regulation, no current use of hypoglycemic agents (α-glucosidase inhibitors, canagliflozin), and no history of use of some traditional Chinese medicines such as *polygala tenuifolia* and *senega syrup* therapy. In addition, those with chronic renal failure, chronic liver dysfunction, and acute infection were also excluded from the study. This study was approved by the Ethics Committee of Shanghai Jiao Tong University Affiliated Sixth People’s Hospital, and was in accordance with the 1964 Helsinki declaration and its later amendments or comparable ethical standards. Informed consent was obtained from all individual participants included in the study.

### Anthropometric and biochemical assessments

All participants received a comprehensive physical examination, including height, body weight, and blood pressure. BMI was calculated as weight/height^2^ (kg/m^2^). Blood samples were obtained from all participants in the morning after a 10-h overnight fast to measure the levels of FPG, HbA_1c_ and 1,5-AG. A 75-g OGTT was administered to each patient to assay the 2hPG. Standard laboratory measurements were performed^22^. Serum 1,5-AG levels were measured by an enzymatic method (GlycoMark; GlycoMark Inc., New York, NY, USA) on a 7600 autoanalyzer (Hitachi, Tokyo, Japan) with inter- and intra- assay coefficients of variation (CVs) of <3.5% and <2.5%, respectively.

### Definition and diagnostic criteria

According to the 2010 ADA criteria^23^, those who meet the diagnostic criteria of FPG ≥ 7.0 mmol/L, 2hPG ≥11.1 mmol/L, or HbA_1c_ ≥ 6.5% should be diagnosed with diabetes. FDR indicates the participant had at least one first-degree relative (parent, child, or sibling) with diabetes^24^.

### Statistical analysis

SPSS version 19.0 (SPSS, Inc., Chicago, IL, USA) and MedCalc version 15.2 were used for statistical analysis. Based on a normality test, normally distributed data are presented as mean ± standard deviation values, and skewed data are presented as median with interquartile ranges. Categorical variables are presented as percentages (%). Unpaired Student’s *t* test and Chi-squared test were carried out for inter-group comparisons of normally distributed data, and the Wilcoxon rank sum test along with the Kruskal-Wallis test were used for inter-group comparisons of non-normally distributed variables. Spearman correlation analysis and multiple stepwise regression analysis were conducted to identify independent factors. The ROC curve was generated to analyze the value of serum 1,5-AG in screening diabetes. The optimal cut-off point was confirmed based on the Youden index. A two-tailed *P* value < 0.05 was considered to be statistically significant.

### Ethical approval

This study was approved by the Ethics Committee of Shanghai Jiao Tong University Affiliated Sixth People’s Hospital, and was in accordance with the 1964 Helsinki declaration and its later amendments or comparable ethical standards.

### Informed consent

Informed consent was obtained from all individual participants included in the study.
